# Relationship between static postural control and the level of functional abilities in children with cerebral palsy

**DOI:** 10.1590/bjpt-rbf.2014.0056

**Published:** 2014

**Authors:** Sílvia L. Pavão, Gabriela S. Nunes, Adriana N. Santos, Nelci A. C. F. Rocha

**Affiliations:** 1 Programa de Pós-graduação em Fisioterapia, Universidade Federal de São Carlos (UFSCar), São Carlos, SP, Brasil; 2 Curso de Fisioterapia, UFSCar, São Carlos, SP, Brasil; 3 Departamento de Fisioterapia, UFSCar, São Carlos, SP, Brasil

**Keywords:** cerebral palsy, postural balance, children, functionality, PEDI, rehabilitation

## Abstract

**Background:**

Postural control deficits can impair functional performance in children with cerebral palsy (CP) in daily living activities.

**Objective:**

To verify the relationship between standing static postural control and the functional ability level in children with CP.

**Method:**

The postural control of 10 children with CP (gross motor function levels I and II) was evaluated during static standing on a force platform for 30 seconds. The analyzed variables were the anteroposterior (AP) and mediolateral (ML) displacement of the center of pressure (CoP) and the area and velocity of the CoP oscillation. The functional abilities were evaluated using the mean Pediatric Evaluation of Disability Inventory (PEDI) scores, which evaluated self-care, mobility and social function in the domains of functional abilities and caregiver assistance.

**Results:**

Spearman's correlation test found a relationship between postural control and functional abilities. The results showed a strong negative correlation between the variables of ML displacement of CoP, the area and velocity of the CoP oscillation and the PEDI scores in the self-care and caregiver assistance domains. Additionally, a moderate negative correlation was found between the area of the CoP oscillation and the mobility scores in the caregiver assistance domain. We used a significance level of 5% (p <0.05).

**Conclusions:**

We observed that children with cerebral palsy with high CoP oscillation values had lower caregiver assistance scores for activities of daily living (ADL) and consequently higher levels of caregiver dependence. These results demonstrate the repercussions of impairments to the body structure and function in terms of the activity levels of children with CP such that postural control impairments in these children lead to higher requirements for caregiver assistance.

## Introduction

Children with cerebral palsy exhibit motor disorders that are characterized by uncoordinated movements, maladaptive muscle length changes and postural control dysfunction^1^ ^,^ ^2^. Postural control ensures the proper positioning of the body in space and maintains stability and body alignment by maintaining the projection of the center of pressure (CoP) within the limits of the support base^3^ ^,^ ^4^.

Children with CP experience difficulties with controlling the body's position in space, performing anticipatory adjustments for executing functional activities and reacting to unexpected perturbations of balance^3^ ^,^ ^5^ ^,^ ^6^. The primary causes of the deficits in fine posture control adjustment in children with CP include the order of muscle recruitment, the rate of agonist/antagonist co-activation, incoordination of joint segments and recruitment of fewer motor units responsible for coordinating postural responses^6^ ^-^ ^8^. Together, these changes increase the difficulty of controlling the body in space and define low levels of coordination between the joint segments of the body^9^. Therefore, the child with CP presents with increased CoP oscillation to achieve adaptive success in their daily activities^4^. Because of the neuromotor deficits and biomechanical changes in these children, the alignment between body segments and CoP maintenance within the limits of the support base are compromised^9^ ^,^ ^10^.

Therefore, the set of dysfunctions that are manifested in CP can affect postural control while standing and thus compromise functional activities. This inference has been reinforced by the findings reported in some studies, which have claimed that these children might face difficulties with maintaining upright posture maintenance and performing activities of daily living (ADLs)^3^ ^,^ ^11^, thus reducing their level of independence^3^ ^,^ ^6^ ^,^ ^8^ ^,^ ^12^ ^,^ ^13^.

In addition, studies have reported functional consequences of these deficits in postural control mechanisms on the gait^11^ ^,^ ^12^ and reach of children with CP^14^ ^,^ ^15^ and have revealed difficulties in situations that involve rapid changes in load bearing^16^ and unexpected disruptions in upright posture^9^ ^,^ ^17^ required to perform some activities of daily living.

Although the literature has highlighted an apparent relationship between postural control and functionality^3^ ^,^ ^15^, in a database search, no studies were found that correlated static postural control with the functional ability levels displayed by children with CP. Knowledge of this relationship demonstrates the importance of maintaining stability and alignment between the body segments in an upright posture while performing functional activities. Additionally, this knowledge allows one to extrapolate force platform data analyses to these children's activities of daily living.

The literature describes instruments for evaluating functional abilities such as the *Pediatric Evaluation of Disability Inventory *(PEDI), which has been reliably standardized and validated for the Brazilian population^18^ ^,^ ^19^. This evaluation tool enables investigators to measure functionality based on activities that children would normally perform during their daily routines and to measure their dependence on a caregiver^20^.

Given the potential of PEDI to assess the functional abilities of children with CP by using activities similar to those performed in their daily routines and the lack of studies that have correlated static postural control and the functional ability level in this population, it was believed that studies examining this relationship could demonstrate the direct consequences of deficits in postural control on the level of independence of children with CP in terms of activities involving mobility, self-care and social function.

Therefore, the present study aimed to investigate the relationship between static postural control while standing and the functional ability levels of children with CP.

## Method

### Participants

The present study was essentially a cross-sectional study with an applied aspect. Children of both genders who had been diagnosed with spastic CP at levels I and II of gross motor function (GMFCS)^21^ and who were between 5 and 12 years of age were selected. It has been reported that in this age range, sensory integration for postural control is enhanced by improvements in sensory integrative mechanisms and increased vision contributions^22^, thus allowing the generation of responses that ensure successful balance maintenance.

Children were required to be able to follow the simple commands needed to perform the proposed tasks and to maintain an upright posture without support for at least 30 seconds. Parents or guardians were required to sign informed consent (IC) forms for their children to participate in the study. Children who exhibited lower limb muscle shortening that would limit their function in an upright position, such as shortening of the hamstrings, adductors, hip flexor or plantar flexor muscles, were excluded. Children who had undergone orthopedic surgery of the lower limbs in the last 12 months or had received botulinum toxin injections in the last 6 months were also excluded.

### Procedures

This study was submitted to the National Council of Health and the Ethics Committee on Human Research of the Universidade Federal de São Carlos (UFSCar), São Carlos, SP, Brazil and was approved (Opinion No. 490/2010). After the informed consent form was signed, the children underwent an initial assessment to collect anthropometric and orthopedic data.

Postural control for each child was then evaluated while in a standing position. A Bertec 400 force platform (EMG Sistem do Brasil^(r)^, São José dos Campos, SP, Brazil) was used at an acquisition frequency of 100 Hz. The children were asked to remain standing on the force platform with their feet parallel to and aligned with the sides of their hips^7^. The initial foot position was marked to ensure consistency throughout the trials. While standing on the platform, the children were asked to remain as still as possible for 30 seconds while looking at a circular figure positioned 1 meter in front of them at eye level. Each child performed the task a total of 5 times (2 adaptation and 3 valid attempts). Each trial was separated by a rest period of 120 seconds^23^.

An evaluation of each child's functional abilities was then performed using the PEDI instrument, which had been standardized and validated for the Brazilian population^19^. This instrument evaluates children between the ages of 6 months and 7.5 years. However, given that the children evaluated in this study had functional abilities compatible with the age limits determined by the PEDI, we evaluated children older than the upper age limit indicated by the instrument. Accordingly, it was not possible to use the normative score calculations and therefore only the raw scores for each child were used^2^.

The PEDI is divided into 3 distinct parts that provide information about 3 different areas of functional performance. The first part evaluated the functional abilities of children in the areas of self-care, mobility and social function. Each of the 197 evaluated items received a score of 1 if the child performed the function or 0 if he/she could not perform it. The second part quantified the assistance provided by the caregiver to the child while performing self-care (8 items), mobility (7 items) and social function (5 items) tasks. In this second part, each item is evaluated on an ordinal scale from 0 to 5, where 0 indicated a need for maximum assistance and 5 indicated independence. The sum of the scores yielded a total raw score for each of the 3 areas of functional abilities. Therefore, a higher score corresponded to better functional performance for the child. The third part of the PEDI provided information about the modifications necessary to perform functional tasks in the previously described 3 areas (self care, mobility & social function).

For the present study, only data from the first 2 parts of the instrument, functional abilities and caregiver assistance, were used from among the 3 evaluated domains (self-care, mobility and social function)^20^. For the scale application, the evaluator was trained and achieved an intraobserver agreement index of 85%.

### Data analysis and statistics

Data were captured from the kinetic force platform analysis using Bertec software (Bertec Corporation, Columbus, OH, USA), and data analysis and dependent variable calculations were performed by implementing routines in the MATLAB software environment (Mathworks Inc., Natick, MA, USA). Data were normalized according to the children's weights^7^. The force platform data were filtered in MATLAB software, using a fourth-order low-pass Butterworth digital filter with a cutoff frequency of 5 Hz.

The kinetic variables of static postural control analyzed in the present study while standing were the anteroposterior amplitude of the CoP displacement (Amp AP; in cm), the mediolateral amplitude of the CoP displacement (Amp MLin cm), the CoP oscillation area (Area; c^2^) and d) the velocity of the CoP oscillation (Vel in cm/s)^24^.

The dependent variables of the PEDI instrument were the raw instrument scores in the functional abilities area for the domains of self-care (FAsc), mobility (FAmob) and social function (FAsf) and in the caregiver assistance area for the domains of self-care (CAsc), mobility (CAmob) and social function (CAsf).

Descriptive results were generated by calculating the means and standard deviation. For the statistical data analysis, the mean of the 3 trials performed on the force platform for each of the variables was used. The Shapiro-Wilk test confirmed the absence of normality in the data distribution (p≥0.05).

Spearman's correlation was used to study the relationship between the mean of the 3 attempts while standing on the force platform and the PEDI instrument scores because the data were not parametric. The rating value (r) proposed by Munro^25^ was used as a base. A correlation was performed between the variables, and the coefficient of determination was also calculated. The significance level was set at 5% for all analyses. SPSS software, version 10.0 (SPSS Inc., Chicago, IL, USA) was used for the analysis and to generate graphs.

## Results

The study included 10 children with spastic CP (M [media]=9; ±4.9), 5 males and 5 females, including 7 children with spastic hemiplegic CP and 3 with spastic diplegic CP. The results for all correlations are shown in Table 1.

**Table 1:** Spearman correlation (r) values for the standing static permanence variables: antero-posterior amplitude of CoP displacement (Amp AP), medio-lateral amplitude of CoP displacement (Amp ML), area of CoP oscillation (Area), mean velocity of CoP oscillation (Vel) and PEDI scores (p<0.05).

	PEDI Functional Skills			PEDI Caregiver assistance		
COP Variables	Self-Care	Mobility	Social Function	Self-Care	Mobility	Social Function
**Amp AP (cm)**	r=–0.19 p=0.6	r=–0.46 p=0.18	r=0.33 p=0.34	r=–0.60 p=0.06	r=–0.58 p=0.07	r=–0.45 p=0.18
**Amp ML (cm)**	r=–0.23 p=0.50	r=–0.55 p=0.09	r=0.22 p=0.54	**r=–0.82** **p=0.003**	r=–0.57 p=0.08	r=–0.42 p=0.21
**Area (cm** ^**2**^ **)**	r=–0.22 p=0.54	r=–0.52 p=0.12	r=0.42 p=0.22	**r=–0.78** **p=0.007**	**r=–0.63** **p=0.04**	r=–0.36 p=0.30
**Vel (cm/s)**	r=–0.28 p=0.43	r=–0.50 p=0.13	r=0.28 p=0.42	**r=–0.70** **p=0.02**	r=–0.62 p=0.06	r=–0.56 p=0.08

The present study found significant and negative correlations between the child's CoP behavior while standing and the functional abilities. There were also strong negative correlations between the variables of the ML amplitude of CoP displacement (r=-0.82, p<0.05), CoP oscillation area (r=-0.78, p<0.05) and mean velocity of the CoP oscillation (r=-0.70, p<0.05) and the self-care domain scores in the area of caregiver assistance. Additionally, there was a moderate negative correlation between the CoP oscillation area and the mobility scores (r=-0.63, p<0.05) in the area of caregiver assistance. The correlation values and coefficients of determination are shown in Table 2.

**Table 2:** Presentation of significant correlations (r), statistic significance (p), correlation classification according to Munro and determination coefficient (%).

VARIABLES	r	P	CLASSIFICATION	%
**Amplitude ML (cm) - ACac**	– 0.824	<0.05	Strong	67
**Area (cm** ^**2**^ **) - Acac**	– 0.784	<0.05	Strong	61
**Velocity (cm/s) - ACac**	– 0.704	<0.05	Strong	49
**Area (cm** ^**2**^ **) - ACmob**	– 0.635	<0.05	Moderate	40

Based on the correlation coefficients shown in Table 2, the results suggest that while standing, 67% of the variability in the Amp ML of the CoP was related to variations in the self-care scores in the area of caregiver assistance, indicating that medial-lateral directional displacement resulted in 67% lower scores in the domain of self-care.

Additionally, 61% of the variability in the CoP oscillation area was related to variations in the scores for self-care in the area of caregiver assistance, indicating that increased and more rapid CoP oscillations were related to 61% lower self-care scores in the area of caregiver assistance. Moreover, 49% of the variability in the velocity of the CoP oscillation appeared to be related to variations in the self-care scores in the area of caregiver assistance, indicating that increased and more rapid CoP oscillations are related to 49% lower self-care scores in the area of caregiver assistance. Finally, 40% of the variability in the CoP oscillation area was related to variations in the mobility score in the area of caregiver assistance, indicating that larger areas of displacement resulted in 40% lower mobility scores.

## Discussion

The aim of the present study was to investigate the relationship between postural control while standing and the functional ability level in children with CP via an analysis of variables related to the CoP behaviors while standing and an evaluation of the child's functional performance according to the PEDI instrument.

The results suggested that in children with CP, higher Amp ML values for CoP displacement and the area and mean velocity of the CoP oscillation correlated with lower scores in the area of caregiver assistance in the self-care and mobility domains of the PEDI.

This relationship meets the provisions of the International Classification of Functioning, Disability and Health (ICF), which define the health status of individuals as multi-directional relationships between different domains of health. Accordingly, changes in body structure and function (such as deficits in postural control as observed in CP) are related to the level of activity and participation^26^. Therefore, the CoP behavior while standing might correlate with a child's reduced performance in functional abilities and lower level of independence from caregivers. Similarly, the deficits that are experienced by these children in performing functional activities may be related to larger CoP excursions while standing by restricting their participation in the environments in which they are placed and limiting their experiences in different postures.

Among the children evaluated in the present study, changes in the body structure and function, which were represented by higher ML CoP displacement values (indicative of less control over the postural adjustments required to maintain balance^27^), correlated with a greater reliance on their caregivers to perform self-care activities, including feeding, bathing, changing clothes, personal hygiene and bathroom use.

Previous studies have reported higher CoP displacement values in the ML direction at different positions for children with CP relative to their typical peers^1^. According to the present results, the larger ML displacement might correlate significantly with the level of functionality in these children (67%), thus reflecting a deficit in maintaining proper oscillation while performing functional tasks as measured with the PEDI instrument. The other 33% might be related to the changes in tone or muscle strength that were commonly observed in these children.

The relationship between greater ML displacement and lower self-care domain scores can be explained by the strategies employed by this population to maintain balance. Typical children preferentially use an ankle strategy to prevent imbalances and potential falls^4^ ^,^ ^9^. However, because of their neuromotor disorders, children with CP have deficiencies in recruiting the muscles around the ankle joint^4^ ^,^ ^9^ ^,^ ^23^. Therefore, children with CP preferably use muscles around the hip^9^, a strategy that is associated with larger ML displacements^23^.

Therefore, neuromotor deficits such as changes in the order of muscle recruitment and the loss of interarticular coordination in children with CP^9^ appear to be associated with a greater dependence on caregivers for the performance of certain activities, including bathing, brushing teeth, changing clothes and hair care. These activities are commonly performed while standing, the position in which the body is most intensely subjected to destabilizing forces.

Although it is essential for maintaining postural control while standing^23^, the Amp AP of the CoP displacement did not significantly correlate with the children's level of functionality in any of the evaluated PEDI domains. It is possible that displacements in this direction did not exhibit significant variability in the children evaluated and thus it was not possible to find a correlation. These children exhibited greater oscillations in the ML direction. These results can be explained by the preference of children with CP for using the hip strategy, which is reflected by the larger ML displacements, to maintain balance^23^. Because these children are less able to activate the muscles around their ankles, they recruit the hip muscles instead to maintain stability^9^.

Additionally, children with higher CoP oscillation areas had lower scores in the domains of self-care and mobility. Larger CoP oscillation areas indicate reduced control over the body in response to body imbalance^17^ ^,^ ^28^. The performance of self-care and mobility activities involves reaching for and manipulating objects as well as deliberately moving the CoP^4^ ^,^ ^7^ ^,^ ^15^, which generates internal forces that produce instability and alter the alignment between the body segments. Therefore, in the present study, postural control was associated with independence in self-care in 61% of the children and independence in mobility in 40% of the children. As noted, other factors that were not evaluated in this study might also be related to the independence of children with CP in terms of self-care and mobility activity performance.

Children with CP have larger CoP oscillation areas while standing^28^. These larger oscillation areas represent a better exploitation of the support base to generate successful postural adjustments and avoid imbalance. Because of their neuromotor deficits, children with CP experience more difficulty in coordinating their responses to imbalance and thus must more broadly exploit their support bases. This increased exploitation is intended to attract a greater number of proprioceptive afferents with which to adjust the body positioning in space^15^ ^,^ ^27^ ^,^ ^29^.

In the present study, the deficits in postural control regulation observed in children with CP, which were represented by a greater oscillation area, were related to lower levels of functionality, which were represented by a greater reliance of the child on a caregiver for mobility task performance. Studies have indicated that in children with CP, postural control, which is influenced by the child's neuromotor disability severity^5^ and biomechanical constraints^30^, is directly related to independent locomotion^12^. Therefore, in clinical practice, balance control training while standing might represent a method by which children can gain independence in performing tasks requiring locomotion.

This study also revealed that children with higher mean velocities of oscillation had an increased dependence on others to perform self-care activities. The velocity of CoP oscillation is a primary predictor of body stability in postural control analyses and has been inversely correlated with the control of body segments in space^3^ ^,^ ^28^. Therefore, increased velocities reflect reduced control over postural responses, whether through neuromotor deficits or biomechanical changes^30^. Accordingly, the deficits in postural control observed in children with CP result in an increase in postural instability while standing^23^, in which the vast majority of functional self-care activities are performed.

In general, the results of the present study suggest that the difficulties experienced by children with CP when controlling body positioning in space, which result from deficits in body structure and function, influence other domains of a child's health status and interfere with his/her activity levels and social participation^26^.

By becoming more dependent on a caregiver to perform daily routine tasks and by experiencing limited mobility, these children have a limited ability to explore the environment around them and establish social ties with people outside of their families. The continued requirement for help when performing certain tasks prevents children from independently participating in many activities and causes them to become dependent on their caregivers even for social integration.

Because changes in postural control may determine functional changes, increasing postural stability should be a goal of physical therapy programs for children with CP, as physical rehabilitation can improve their level of functionality and thus yield increases in their levels of social interaction with their surrounding environment. Similarly, while considering the results of the present study and the broad and comprehensive definitions of an individuals health characteristics as advocated by the ICF, children with CP should be encouraged to more effectively participate in their own daily routine activities and to become as independent as possible to help them achieve greater postural stability while standing.

According to the new World Health Organization rules established by the ICF, clinical practices should increasingly seek to address the health status of individuals in a more broad and comprehensive manner rather than simply focusing on dysfunctions related to body structure and function. Instead, clinicians should seek to emphasize the impact that these deficits have on the activities performed by individuals as part of their social participation^13^.

Therefore, given the relationship between postural control and the level of functional abilities in children with CP, more studies will be needed to determine the therapeutic approaches that will be most effective in promoting increased functionality and social participation in this population. It is believed that establishing this relationship will generate new directions for clinical practice and thus will guide treatment strategies for balance disorders that will help children to acquire specific functional abilities and establish independence from their caregivers.

## Conclusion

In children with CP, postural control while standing has an important relationship with the level of functionality and thus establishes a direct relationship with the level of dependence on a caregiver. Therefore, the increased difficulty experienced by a child while maintaining a stable standing posture is related to their ADL performance and level of caregiver dependence.
